# Attack Graph Modeling for Implantable Pacemaker

**DOI:** 10.3390/bios10020014

**Published:** 2020-02-19

**Authors:** Mariam Ibrahim, Ahmad Alsheikh, Aseel Matar

**Affiliations:** 1 Department of Mechatronics Engineering, German Jordanian University, Amman 11180, Jordan; 2 Department of Natural Sciences and Industrial Engineering, Deggendorf Institute of Technology, 94469 Deggendorf, Germany; a.alsheikh@gju.edu.jo; 3 Department of Biomedical Engineering, German Jordanian University, Amman 11180, Jordan; As.Matar@gju.edu.jo

**Keywords:** pacemaker, threat modeling, internet of things (IoT) medical devices, vulnerabilities

## Abstract

Remote health monitoring systems are used to audit implantable medical devices or patients’ health in a non-clinical setting. These systems are prone to cyberattacks exploiting their critical vulnerabilities. Thus, threatening patients’ health and confidentiality. In this paper, a pacemaker automatic remote monitoring system (PARMS) is modeled using architecture analysis and design language (AADL), formally characterized, and checked using the JKind model checker tool. The generated attack graph is visualized using the Graphviz tool, and classifies security breaches through the violation of the security features of significance. The developed attack graph showed the essentiality of setting up appropriate security measures in PARMS.

## 1. Introduction

Implantable therapeutic tools are becoming progressively interdependent through the internet of things (IoT) in order to audit vital signs and improve patients’ quality of life. Yet, the IoT imposes major vulnerabilities with such interconnection, and any disturbance could cause significant destruction or life-impeding demands [1,2]. An adversary may construct various attacks to jeopardize both IoT implantable therapeutic equipment and networks [3]. Table 1 illustrates some recent cyberattack incidents that occurred in the medical field. Thus, it is not easy to design and protect medical devices that are able to cope with equipment failures and connectivity and operating systems faults [4]. Security and privacy concerns should also be considered, such as identification, data integrity, confidentiality, authentication, and user and service privacy [5]. A recent survey [6] studied over one hundred medical tools to consider their protection worries with a focus on reported cyberattacks including tampering, sniffing, and unauthorized access. The survey also studied available mitigation methods to handle these worries.

Attack graphs provide a viewable technique to determine risks within interoperable systems. The actions needed to conduct an attack can be identified utilizing this technique. The identification of attacks helps engineers to establish defensive actions in order to eliminate the execution of an attack [14]. For instance, a method is presented by [15] for indicating the best placement of a collection of IoT tools within an institution using a traditional attack graph which is augmented to consider the substantial placement of IoT tools and their connectivity effectiveness.

Attack graphs can also help forensic investigators to identify many possible attack paths. An empirical study is provided by [16] on the growth of using data gathered by smartphone tools (developed to correlate a therapeutic tool) as digital clue in legal cases. A report is included about evidence which is possibly helpful in a digital forensics inspection.

A digital inspection system is proposed by [17] for the examination of fatal attack scenarios on cardiac implantable medical devices (IMDs). The system reports the identification and regeneration of possible attack scenarios that result in a patient’s death. An approach of three stages is proposed, along with a collection of approaches to use in every stage. In the first stage, the approach aids determining the reason for a death based on the therapeutic conclusions gathered by the IMD. Second, the approach follows the entries and system logs gathered from the IMD under consideration, which determine the critical actions associated with distant access and construction. The technique aims to collect the possible attack scenarios that could achieve similar impact in the gathered log proof, as if they had been conducted. A library of threats and a model checking established algorithm are utilized to conduct the automatic reformation which is made in forward chaining. The third stage of the approach correlates the generated scenarios, identifies the most persuasive composite of medical and vocational scenarios, and confirms the presence of abnormal attitude in the chosen composite that caused a patient’s death.

The main contribution of this work manifests an approach for developing attack graphs for the pacemaker automatic remote monitoring system (PARMS). This demands a general specification of system model (design and communications, units, resources, protections, vulnerabilities, and attack instances), and exploration of the security concerns. The model and the security properties are encoded using architecture analysis and design language (AADL) [18] and verified using JKind checker embedded software [19]. The developed attack graph contains the attack scenarios causing system compromise through gaining ability to alter the settings of the home monitoring device. Thus, controlling the wireless pacemaker and jeopardizing the patient’s life. The resulting graph is visualized utilizing Graphviz [20]. The rest of this paper is organized as follows: Section 1.1 reviews the relevant work. Section 2 presents the modeling process of the pacemaker automatic remote monitoring system (PARMS). Section 3 illustrates attack graph construction and visualization for the PARMS. Section 4 recaps and discusses some forthcoming work.

### 1.1. Related Work

Different papers were investigated in the literature for modelling attack graphs for medical devices. A model-based system, a safety and security co-engineering (MB3SE) technique, and a correlated toolchain for the implementation of medical equipment was proposed by [21]. The toolchain included architecture modelling and safety and cyber-security risk analysis tools. Explanations for security concerns of 5G networks aiding electronic healthcare applications were presented by [22]. The explanations incorporated knowledge graph development, automated attack and protection technologies, and a security testbed.

An approach is presented by [23] for developing attack trees for IMDs which receive two inputs: functional workflow and a hazard study of the IMD in consideration. A process-modeling software is utilized to illustrate the IMD system as it is arranged, booted up, and managed by the caregiver. Hazards can be identified as system states that are built-in unprotected for the user. Hazard study requires determining system states that will ultimately cause critical harm to the patient.

Threat modeling is examined in medical cyber physical systems (MCPS) by [24]. This includes the roles of stakeholders and system components, trust models, threat models, and threat analysis. An abstract architecture is also sketched for an MCPS to demonstrate various threat modeling options.

A methodology has been developed by [2] for generating attack trees for patient controlled analgesia (PCA-IMD). This process contains four steps: (1) process modeling, (2) fault tree analysis (FTA), (3) attack tree generation, and (4) quantification. First, the user of the PCA-IMD takes a depiction of the workflow of the PCA-IMD and constructs a process-modeling design for it. Once the process model is constructed, the IMD user establishes the distinct hazards that can happen as a result of running the system, leading to extra infusion.

Two internal activities are studied by [25], involving the utilization of Universal Serial Bus (USB) drives and Compact Disc Read-Only Memory (CD-ROM) as the entrance methods leading to data loss in the healthcare firm surroundings. The generated augmented threat trees show the vulnerabilities abused, the actions required to abuse them, and the fingerprint implemented by the attackers’ functionalities. A Markov models set is developed by [26] for a healthcare IoT foundation, that enables the consideration of the particularity of clients’ machines, connectivity, advancement of data stream, and protection and security worries of these elements.

The modeling and study of cyberattacks utilizing a multimodal graph technique is shown by [27]. This work illustrates how cyber actions, parties, targets, and networks that gathered them can be modeled using a multimodal graph, such that multiple graphs of distinct modalities are connected to show the features of the attack.

A framework is presented by [28] for modeling and assessing security of the IoT which incorporates preprocessing, security model generation using a hierarchical attack representation model (HARM), conception and repository, security study, and transformations and updates. In the scheme, an IoT, security model generators, and an evaluator are implemented.

The authors of [29] investigated whether the ideas of model checking and attack tree refinement correspond to using an IoT healthcare illustrative example. The extension by model checking and the enclosing of attack trees into the Isabelle internal scheme permitted the investigation of this correspondence utilizing the analytical strict and automated proof assistance of Isabelle. Hence, reassessing the interpretation of state evolution in model checking and importing a variation that showed the attack sequences. This permitted the conversion of attack paths established by model checking into the attack tree refinement procedure.

An attack graph-based study is presented by [4] of attacks on a certain interoperability surrounding to provide patient pain medication (PCA) among multiple levels of interoperability from simple data gathering to complete closed loop control. Explanations of the potential prevention methods are determined for every class of attack vectors. The work showed that security has a deep impact on the safety of medical device interoperability and the patients they are provided to.

Conceptual graphs are collected by [30] with Dung’s disputation system that supplied convenient extensions for dependable selection procedures, all adapted to telemedicine in general and tele-expertise in particular. The work implemented the visual graph of attacks where distinct interpretation of the reasoning logic is adapted to verify the possible adequate arguments.

A systematic threat-modeling approach is proposed by [31] to investigate IMD security. The attack tree approach provided an overall and organized scheme of the strengths and weaknesses of the IMD system. The work showed a systematic method for conducting system-level security examination to incorporate various potential attack surfaces. The research done by [14] demonstrated attack graph modeling on hypothetical ambulatory medical equipment. The research examined specific attacks that jeopardized ambulatory equipment, like physical attacks and social engineering.

## 2. Pacemaker Automatic Remote Monitoring System (PARMS) Modeling

In this work, the pacemaker automatic remote monitoring system (PARMS) is modeled to illustrate how hacking into the pacemaker’s system imposes life-threatening risks to patients. The model includes system topology, possible attack instances, and the system’s formal description.

### 2.1. PARMS Topology

**Figure 1:**
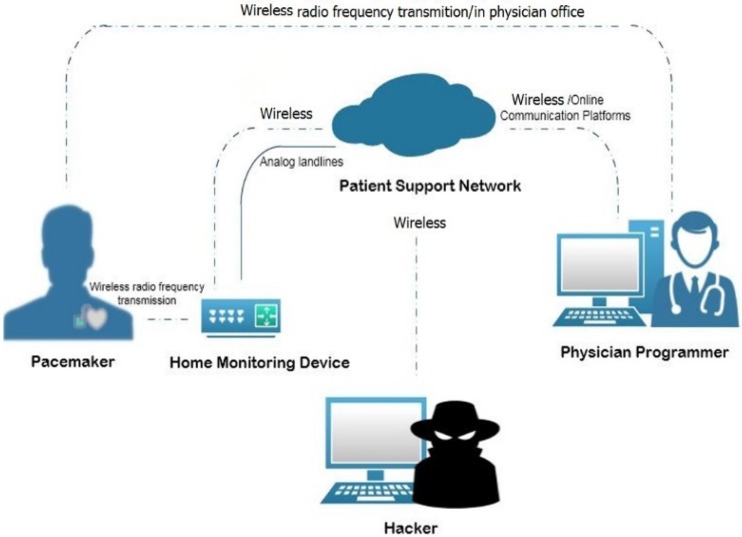
pacemaker automatic remote monitoring system (PARMS).

Figure 1 shows a modified pacemaker automatic remote monitoring system (PARMS) from [32]. The PARMS includes the following components:

- **Wireless Pacemaker**: This is a battery-powered implantable device that produces an excitatory wave at an appropriate site within the heart. The pacemaker initiates the electrical depolarization cycle of the heart at approximately 72 beats/min in the atrioventricular (AV) node to replace a malfunctioning AV node. The AV node is the electrical connecting point from the atria to the ventricles, which continues excitation beyond a partial or total heart block. Modern pacemakers can also store diagnostic data [33].

The pacemaker consists of a cardiac pulse generator, which is a device with a power source and electronic circuitry that produces the excitatory signal. The pulse generator contains a power source “battery” to power it. It also contains a programmable segment to evaluate the heart function and send the appropriate electric impulse signal from the pulse generator to the heart [33]. Other components of the pacemaker are the leads which conduct electrical signals from the pulse generator to the tissue and, in some pacemakers, also conduct signals from the tissue to the pulse generator.

The pacemaker has also an electrode which is located at the end of the lead which continuously measures the heart rate. The sensed signal is then amplified using a low noise amplifier. Next, the amplified signal is filtered using a second order low pass filter, and the result is an appropriate electrocardiogram (ECG) signal. This signal is then applied to a comparator which compares it to a defined threshold to detect the heartbeat event executed by the heart and generates a pulse with every heartbeat using a pulse generator [34]. The wireless pacemakers are embedded with a micro-antenna to enable wireless communication with the home monitoring device and the programmer [35].

- **Physician Programmer (PP)**: A computer with specific software and associated hardware modifications is used to program the pacemaker at the time of implantation. It is used to test pacemaker functionality in the follow-up visits to the physician’s office by sending instructions to change therapy parameters, and reading battery status and heart rhythms. The physician programmer contains a transceiver which communicates with the pacemaker antenna via radio frequency (RF) [32,35].
- **Home Monitoring Device (HS)**: This is a transceiver placed in proximity to the patient and used to monitor the pacemaker by collecting the data that the pacemaker sends periodically at a set of frequencies (e.g., every two days or every week) using RF transmissions via the micro-antenna. The HS then sends data to the physician programmer via analog landlines or wireless data networks. The data incorporate heart rate, battery status, pacing lead impedance, episodes of arrhythmias, conveyed antitachycardia pacing, percent pacing, histogram, real-time and magnet electrograms (EGM), reserved EGMs, arrhythmia reviews, and mode switch period [35,36].
- **Patient Support Network (PN)**: This works on the principle of cloud computing, where the data transmitted from the home monitoring device can be stored into the network servers and can be uploaded onto a secure website that the physician may log in to, to review the data. [32,36].
- **Access Point (AP)**: This exists for outside internet communication. We assume the attacker is located at this point.

Communication among the PARMS’ components can be summarized as follow [32]:

- RF communication between the PP and the wireless pacemaker in order to program it and check its functionality.
- The PP and the PN are connected through online communication platforms: “cloud networking”.
- RF communication between the HS and the wireless pacemaker to inquire different measurements related to the pacemaker such as the battery state and pulse rate.
- The communication between the HS and the PN involves sending measurements to the PN to be stored there and accessed later by the responsible PP. The PN also transmits updates from the network to the HS using analog landlines, a wireless data network, or a wireless Global System for Mobile communications (GSM).

To illustrate how hacking into the pacemaker’s system presents life-threatening risks to patients, two vulnerabilities are identified within PARMS. These are the microprocessor commercial off-the-shelf (COTS) vulnerability, and the firmware update vulnerability due to loss of validation of the source of firmware updates [32]. These vulnerabilities can be exploited resulting in the following possible attack instances:

1. *Intelligent Gathering (IG)*: This is used for gathering information about IoT devices such as Internet Protocol (IP) addresses, and checking the type of firmware, as well as the existence of COTS.
2. *Social Engineering (SE)*: This attack is used to gain access and disclose information. It generally targets enterprises and organizations.
3. *Pivoting (PV)*: This is a standard technique used in penetration testing to navigate from machine to machine [37].
4. *Sniffing (S)*: This attack is used to steal or break off data by capturing the network traffic using a sniffer, for example to get login usernames and passwords that are sent by the HS [32].
5. *Man-in-the-Middle (MiM)*: This attack can occur when the attacker has access over the network connection. Thus, disclosing and manipulating the data flow between two parties.
6. *Phishing (PS)*: This is used to steal user data and allow the attacker to disguise as a trusted entity [38].
7. *Malware Injection (MA)*: The attacker can edit, copy, or install the code at the host. Thus, gaining a root access.
8. *SQL Injection (SQL)*: This attack is used to exploit the web application. Thus, allowing the attacker to gain unauthorized access to the PN database or retrieve information directly from it [39].

### 2.2. Formal Description of PARMS 

The System can be formally described as follows:

1. The attacker is assumed to be located at (AP) and has a root privilege.
2. system components S; variable s ∈ {PP, PN, HS, AP} (static).
3. system connectivity, C ⊆ PP × PN, HS × PN; c_ij_ = 1 if component i is connected to component j (static).
4. System vulnerabilities V; Boolean v_i_ = 1 if vulnerability v ∈ {*COTS*, *firmware*} exists on i (static).
5. Set of possible attacks B; variable b ∈ {IG, SE, S, PS, PV, SQL, MiM, MA}.
6. Attack instances, AI ⊆ B × C; labeled b_ij_ ≡ attack b from source i to target j, b ∈ B.
7. Attacker level of privilege P on HS device; variable p_HS_ ∈ {none, root} (dynamic).
8. Attacker level of privilege PH on host i ∈ {PP, PN, AP}; variable ph_i_ ∈ {none, user, root} (dynamic).
9. Data identification D of component i; Boolean d_i_ = 1 if identification data about i gets collected by attacker (dynamic).
10. Confidential data disclosure K of component i; Boolean k_i_ = 1 if confidential data of component i get disclosed to attacker (dynamic).
11. Data alteration E of component i, Boolean e_i_ = 1 if attacker is able to edit the setting on component i (dynamic).
12. Attack instances pre-conditions:
  - Pre(IG_ij_) = (c_ij_ = 1) ∧ (ph_i_ = root) ∧ (p_HS_ = none)
  - Pre(SE_ij_) = (c_ij_ = 1) ∧ (ph_i_ = root) ∧ (ph_j_ = none)
  - Pre(SE_ij_) = (c_ij_ = 1) ∧ (ph_i_ = root) ∧ (ph_j_ = none)
  - Pre(S_ij_) = (c_ij_ = 1) ∧ (p_HS_ = none) ∧ (d_HS_ = 1) ꓥ (COTS_j_ = 1)
  - Pre(PS_ij_) = (c_ij_ = 1) ∧ (ph_i_ = user) ∧ (d_j_ = 1) ꓥ (firmware_j_ = 1)
  - Pre(PV_ij_) = (c_ij_ = 1) ∧ (ph_i_ = user) ∧ (d_j_ = 1)
  - Pre(SQL_ij_) = (c_ij_ = 1) ∧ (ph_i_ = root) ∧ (ph_j_ = user) ∧ (d_j_ = 1) ∧ (e_j_ = 1) ∧ (k_j_ = 1)
  - Pre(MIM_ij_) = (c_ij_ = 1) ∧ (ph_i_ = user) ∧ (d_j_ = 1) ∧ (e_j_ = 0) ∧ (k_j_ = 1)
  - Pre(MA_ij_) = (c_ij_ = 1) ∧ (ph_i_ = root) ∧ (p_HS_ = none) ∧ (d_j_ = 1) ∧ (e_j_ = 1) ∧ (k_j_ = 1) ꓥ (COTS_j_ = 1 ꓦ firmware_j_ = 1)
13. Attack instances post-conditions:
  - Post(IG_ij_) = (d_HS_ = 1) ∧ (p_HS_ = none)
  - Post (SE_ij_) = (ph_j_ = user) ∧ (d_j_ = 1)
  - Post (SE_ij_) = (ph_j_ = user) ∧ (d_j_ = 1)
  - Post (S_ij_) = (ph_j_ = user) ∧ (d_j_ = 1) ∧ (k_j_ = 1)
  - Post (PS_ij_) = (ph_j_ = root) ∧ (d_j_ = 1) ∧ (e_j_ = 1) ∧ (k_j_ = 1)
  - Post (PV_ij_) = (ph _j_ = user) ∧ (d _j_ = 1) ∧ (k _j_ = 1)
  - Post (SQL_ij_) = (ph_j_ = root) ∧ (d_j_ = 1) ∧ (e_j_ = 1) ∧ (k_j_ = 1)
  - Post (MIM_ij_) = (ph_j_ = root) ∧ (d_j_ = 1) ∧ (e_j_ = 1) ∧ (k_j_ = 1)
  - Post (MA_ij_) = (p_HS_ = root) ∧ (d_HS_ = 1) ∧ (e_HS_ = 1) ∧ (k_HS_ = 1)
14. Initial state: ph_AP_ = root ∧ (∀j ∊ {PP, PN}: ph_j_ = none ∧ p_HS_ = none ∧ (d_j_ = e_j_ = k_j_ = d_HS_ = e_HS_ = k_HS_ = 0)). (Initially, the attacker has a root privilege on access point, no data identification, no confidential data disclosure, and no ability to alter the setting of HS).
15. Security property (*φ*): The attacker has no ability to edit the setting on the home monitoring device HS. Thus, jeopardizing patient’s life. This property can be then described by a computational tree logic (CTL):
  φ ≡ AG (e_HS_ = 0) ≡ AG (¬ (e_HS_ = 1))

## 3. Attack Graph Generation

**Figure 2:**
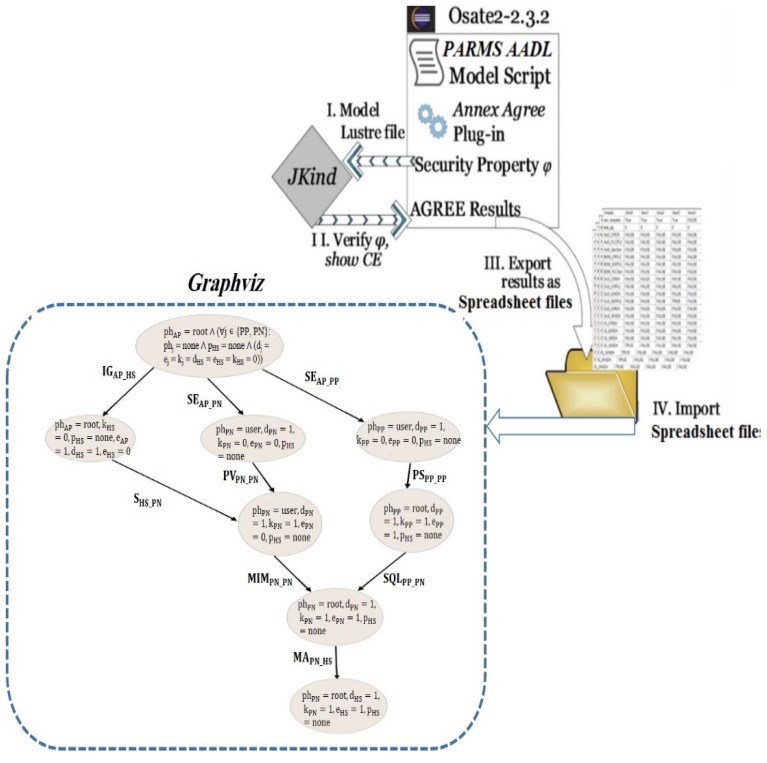
Cyber-attack scenarios implementation workflow. AADL: architecture analysis and design language, CE: counter example.

Two software programs were utilized to conduct the cyberattack scenarios’ generation and visualization, as shown in Figure 2. These tools are JKind model checker and Graphviz. JKind is a software tool that we used to conduct cyberattack scenarios against the PARMS [40]. The model checker keeps checking repeatedly if a given finite-state model of a system meets a given security property of importance. JKind is an infinite-state model checker for analyzing safety attributes of a system asserted in Lustre, a data flow synchronous terminology arranged for programming reactive systems like automatic control and auditing systems [41]. The JKind employs a back-end satisfiability modulo theories (SMT) solver to validate if a system model complies with a specific temporal logic property in every execution of the system. A wrong execution in which a property is not fulfilled is expressed as a counter example (CE) illustrating a sequence of attack instances (i.e., attack scenarios).

The PARMS depiction model of the parts and their interfaces and links is defined using architecture analysis and design language (AADL), within the open-source integrated development environment (Osate2). The AADL model is confined by assume guarantee reasoning environment (AGREE) annex plug-in in which the constants or variables are established locally. The AGREE plug-in translates the AADL+Annex models and properties to Lustre and communicates with JKind which verifies the system against the security property under study φ, and gives the result as a CE.

**Figure 3:**
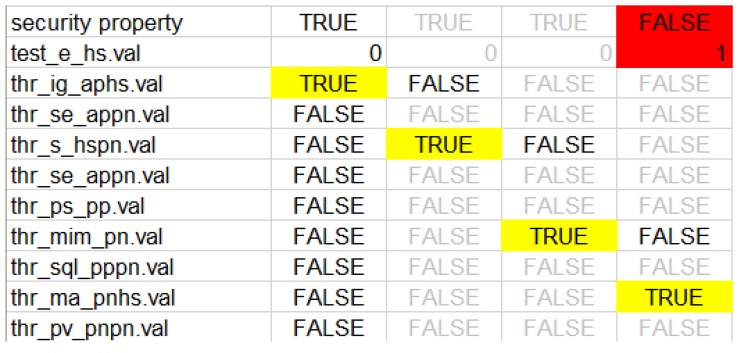
CE1 spreadsheet.

Considering the given security property φ, the goal of the attacker is to gain a root access on the home monitoring device (HS), and therefore gain the ability to alter the settings of the HS. Thus, imposing a life-threatening risk to patient. The JKind model checker generated the following counter example (*CE1*: *IG_AP-HS_* → *S_HS-PN_* → *MIM_PN-PN_* → *MA_PN-HS_*) as a spreadsheet shown in Figure 3.

This attack sequence can be summarized as follows. Initially, the attacker has a root privilege on AP, an *IG_AP-HS_* attack is initialized to gather information about the HS (e.g., IP addresses). After the *IG_AP-HS_* attack an *S_HS-PN_* attack is launched between the HS and PN to get login username and password. This will allow the attacker to access the PN with user privilege therefore disclosing patient and HS information. Using the disclosed information, an *MIM_PN-PN_* attack is launched against the PN components to gain a higher privilege (root privilege). Using this privilege, an *MA_PN-HS_* attack is conducted exploiting a COTS vulnerability in the HS to gain a root access to it. By doing so, the attacker can alter the settings of the HS which will affect the wireless pacemaker and jeopardize the patient’s life.

The generated counter example CE1 is encoded in disjunction with the property φ under study, that is *φ* *∨ CE1*. A new counterexample complies with: *¬ (φ* *∨ CE1) = ¬ φ* *∧ ¬ CE1*, i.e., a counter example of φ distinct from *CE1*. This produces a new counter example (*CE2*: *SE_AP-PN_* → *PV_PN-PN_* → *MIM_PN-PN_* → *MA_PN-HS_*). By continuing this process, three CEs were found, producing the complete attack scenarios (attack graph).

**Figure 4:**
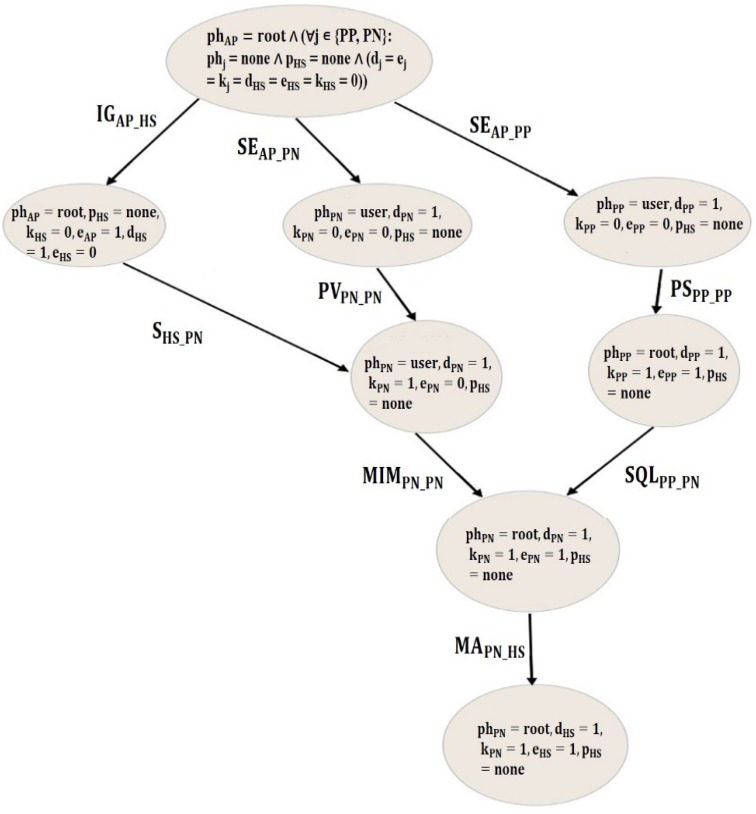
*PARMS* generated attack graph. MA: Malware Injection, SE: Social Engineering, IG: Intelligent Gathering, MiM: Man-in-the-Middle, SQL: SQL Injection, S: Sniffing, PV: Pivoting, PS: Phishing, HS: Home Monitoring Device, PN: Patient Support Network, AP: Access Point, PP: Physician Programmer p_HS_: Attacker level of privilege on HS, ph_i_: Attacker level of privilege on host i, d_i:_ Data identification of component i; k_i_: Confidential data disclosure of component i, e_i_: Data alteration of component i.

In order to visualize the union of generated cyberattack scenarios (attack graph), the Graphviz tool and DOT graph description language are used. Graphviz is a package of open-source tools used to represent structural information as diagrams of abstract graphs and networks. Graphviz takes the descriptions of graphs in a simple text language [20]. The resulting attack graph shown in Figure 4 consists of arrows and nodes. Each arrow illustrates a possible occurrence of an attack instance, while each node represents the system state resulting from executing the attack instance. An attack scenario is a sequence of attack instances represented by any path from the initial node to the final node in the attack graph. The shown attack graph has three attack scenarios that terminate in a reachable state where the settings of HS can be altered by the attacker. Hence, the attacker can gain a root privilege on the pacemaker, which may threaten the patient’s life.

The generated graph may aid system administrators to decide the placement of appropriate detection and prevention measures. For instance, experimental results showed that an MA attack can never be correctly conducted against the HS without running MIM or SQL attacks first against the PN. Thus, by way of preventing MIM and SQL, the system administrators can also eliminate the MA attack which would immensely enhance the system security.

In addition to that, the MA attack against the HS required exploiting the COTS vulnerability in its operating system or the firmware update vulnerability. Therefore, securing the HS operating system and deploying an intrusion detection system (IDS) between the HS and the PN may prevent the attacker from executing the remaining attacks.

The feasibility of protecting implantable medical devices (IMDs) is explored by [42] without adjusting them by carrying out security strategies completely on an external device called a shield. The shield is placed between the IMD and possible correspondents, e.g., worn on the body close to the implanted device. The shield performs as a gateway that conveys messages between the IMD and accredited endpoints. Such an approach improves the security of IMDs for patients who already have them and enables medical staff to access a protected IMD by discarding the external device or turning it off.

## 4. Conclusions

In this work, attack graph generation for PARMS is presented using a JKind model checker and DOT language within Graphviz. The idea for modeling is the application of an architectural descriptive language to capture the security-related details of PARMS that an attacker may exploit to impose life-threatening risks to patients. The main goal of this research is to increase the awareness about the security of IoT medical devices. This is done by identifying some of the cyberattacks and estimating their impacts against PARMS. Cyberattacks and vulnerabilities need to be taken into consideration when designing medical IoT devices. Even though some healthcare companies aim to consider within their product development life-cycle safety and security concerns, yet more testing and verification methods are required to produce a systematic method to test the detection or mitigations determined or provided during the safety and security analyses phase against some sophisticated hacking tools. It is important that attacks and defenses be carefully and independently investigated in order to accurately assess risk of the attack and effectiveness of the defense. Determining appropriate detection and mitigation techniques are future directions to pursue.

## Figures and Tables

**Table 1:** Cyberattack incidents in the medical field.

Date	Country	Name	Description
August 2011	United States	Medtronic insulin-delivery system	Hacked the insulin pump and completely disabled it [7]
2008	United States	Cardiac defibrillator	Hacked cardiac defibrillatorto change the device’s settings, ordering it to deliver a shock, and disabling it [7]
2017	United Kingdom	16 United Kingdom hospitals	Freezing systems and encrypting files [8]
2014	United States	Boston Children’s Hospital	Caused the hospital network to lose internet access using distributed Denial of Service (DoS) attack [9]
January, 2015	United States	Anthem	Breached a database with 80 million customers records [10]
July, 2018	England	National Health Service (NHS)	A data breach caused the NHS to share confidential health data of 150,000 patients [11]
June, 2018	Singapore	SingHealth	The data of 1.5 million patients were stolen [12]
2019	United States	NeuroSky 156 brain–computer interface application	Victims’ brain wave data were stolen [13]
